# Epstein-Barr Virus Stimulates Torque Teno Virus Replication: A Possible Relationship to Multiple Sclerosis

**DOI:** 10.1371/journal.pone.0032160

**Published:** 2012-02-22

**Authors:** Silvia S. Borkosky, Corinna Whitley, Annette Kopp-Schneider, Harald zur Hausen, Ethel-Michele deVilliers

**Affiliations:** 1 Division for the Characterization of Tumorviruses, Deutsches Krebsforschungszentrum (DKFZ), Heidelberg, Germany; 2 Division of Biostatistics, Deutsches Krebsforschungszentrum, Heidelberg, Germany; La Jolla Institute for Allergy and Immunology, United States of America

## Abstract

Viral infections have been implicated in the pathogenesis of multiple sclerosis. Epstein-Barr virus (EBV) has frequently been investigated as a possible candidate and torque teno virus (TTV) has also been discussed in this context. Nevertheless, mechanistic aspects remain unresolved. We report viral replication, as measured by genome amplification, as well as quantitative PCR of two TTV-HD14 isolates isolated from multiple sclerosis brain in a series of EBV-positive and -negative lymphoblastoid and Burkitt's lymphoma cell lines. Our results demonstrate the replication of both transfected TTV genomes up to day 21 post transfection in all the evaluated cell lines. Quantitative amplification indicates statistically significant enhanced TTV replication in the EBV-positive cell lines, including the EBV-converted BJAB line, in comparison to the EBV-negative Burkitt's lymphoma cell line BJAB. This suggests a helper effect of EBV infections in the replication of TTV. The present study provides information on a possible interaction of EBV and TTV in the etiology and progression of multiple sclerosis.

## Introduction

The pathogenesis of multiple sclerosis (MS) is probably triggered by a combination of genetic and environmental factors [1]–[5]. Frequent childhood infections, lower socioeconomic status and higher sibling exposure lead to a lower MS prevalence, remarkably similar to observations made in childhood leukemias [6]. Children experiencing viral infections later in life are at risk for developing this disease [1], [3], [4], [7]–[9]. More sensitive detection methods have enabled earlier recognition of pediatric MS, leading to an estimated 2–5% of cases developing before the age of 16 years [10]–[12]. The disease has even been diagnosed in children between 2 and 5 years of age [13], [14].

Several viruses have been considered to be involved [3], [4]. Morbilli- and coronaviruses [15], [16] which, similar to Theiler's murine encephalomyelitis virus (TMEV) in mice [3], [17], induce demyelinating disease in their natural hosts. JC polyomavirus was also proposed, but is presently considered to be a bystander in MS [18], [19]. A number of herpesviruses have been investigated as etiological factors for MS. Activation of varicella zoster virus (VZV) was demonstrated during exacerbations [20], [21]. An upregulation of the endogenous retroviruses HERV-H and HERV-W transcription was mediated by herpesviruses Herpes simplex-1 (HSV-1), human herpesvirus-6 (HHV-6) and VZV [22], [23]. A more direct role for HHV-6 in MS was suspected [24], [25], but was subsequently questioned because no difference was observed in the HHV-6 prevalence between cases and controls [26], [27], although its possible role prior to onset of disease was not excluded [28]. HHV-6 glycoprotein expressed on the surface of infected T lymphocytes allows for its fusion to oligodendrocytes and astrocytes expressing the CD46 receptor which is necessary for viral entry [29]. Cross-reaction of HHV-6 and myelin has also been reported [30]. Individuals with a history of EBV-induced infectious mononucleosis are at 2- to 3-fold increased risk of developing MS [31], [32] in contrast to the risk being extremely low in EBV-negative individuals [33]. Elevated EBV antibody titres have been associated with the onset of MS [4], [9], [10], [12], [34]–[36], but not during the course of disease [37] and whether active EBV infection actually plays a role in ongoing MS disease is controversial [38]–[40]. Mechanistic aspects through which EBV infections may participate in the pathogenesis of MS also remain elusive, despite the compelling epidemiological data available [32], [33].

Infection-induced molecular mimicry has been implicated in several autoimmune diseases including MS [41]–[44]. CD4^+^T lymphocytes responding to the latent EBV nuclear antigen-1 (EBNA-1) are increased in patients with MS [45], [46] and these cross react with myelin antigens [47]. Our previous study [43] demonstrated the response of clonally expanded CD4^+^T cells isolated from an MS patient to the poly-arginine motif present in the open reading frame 1 (ORF1) of certain torque teno virus (TTV) types. Serum samples from this patient, taken during exacerbation and 24 months later, harboured TTV DNA sequences. In addition, we demonstrated TTV DNA in 6 of 13 brain samples, 12 from MS brain and one in HHV-6 encephalitis brain.

The disease-inducing potential of TTV and mechanisms involved in their replication are not understood. TTV was first identified in a Japanese patient suffering post-transfusion hepatitis of unknown etiology [48]. A multitude of TTV genotypes with marked genetic heterogeneity has subsequently been identified [49]. Primary infection occurs very early in life with 47,6% of children infected with at least 3 TTV types at 6 months of age and 93% at 1 year of age [50], [51]. Their genomic diversity and ubiquitous prevalence have posed main obstacles in attempts to define a role for TT viruses in the pathogenesis of disease [49], [52], [53]. TTV DNA has been demonstrated in many organs and tissues [43], [54]–[56]. Peripheral blood act as reservoir for TT viruses [57], [58] and replicative viral DNA has been demonstrated in bone marrow cells and the liver [59], [60]. Other reports have implicated TTV infections in autoimmune diseases [52], [61]–[63].

Influences of host-dependent factors, genotype predilection or environmental triggers on the long-term replication of TT viruses have not been investigated. Short term replication and transcription were described in cell lines of diverse origins [64]–[66]. We recently reported long-term replication of TT viruses with the concomitant formation and independent replication of subviral μTTV (consisting of 10–20% of the mother genome) [67]. We evidenced intracellular viral-like particle formation 7 days after transfection of the TTV-tth8 full-length genome into an Hodgkin's lymphoma cell line L428 [65].

Studies in the past concentrated on single virus infections as etiological factors of MS, whereas interactions between viruses in its pathogenesis have not been approached. Herpesviruses have been known to induce amplification of other viral genomes, as well as cellular sequences [68]–[72]. EBNA-1 enhances replication of Hepatitis C virus (HCV) [73]. The current study analysed the question whether EBV can stimulate or induce TTV replication *in vitro*. We have isolated a series of full-length TTV genomes from diseased brain of MS patients [67]. We transfected two of these TTV isolates into EBV-carrying cell lines. Two EBV-negative cell lines were used as controls. Quantitative PCR analyses clearly demonstrate a helper effect of EBV on the replication of the TTV genome. These results provide additional information for linking viral infections to the pathogenesis of MS.

## Results

### In vitro replication of TTV-HD14b and TTV-HD14c

**Figure 1:**
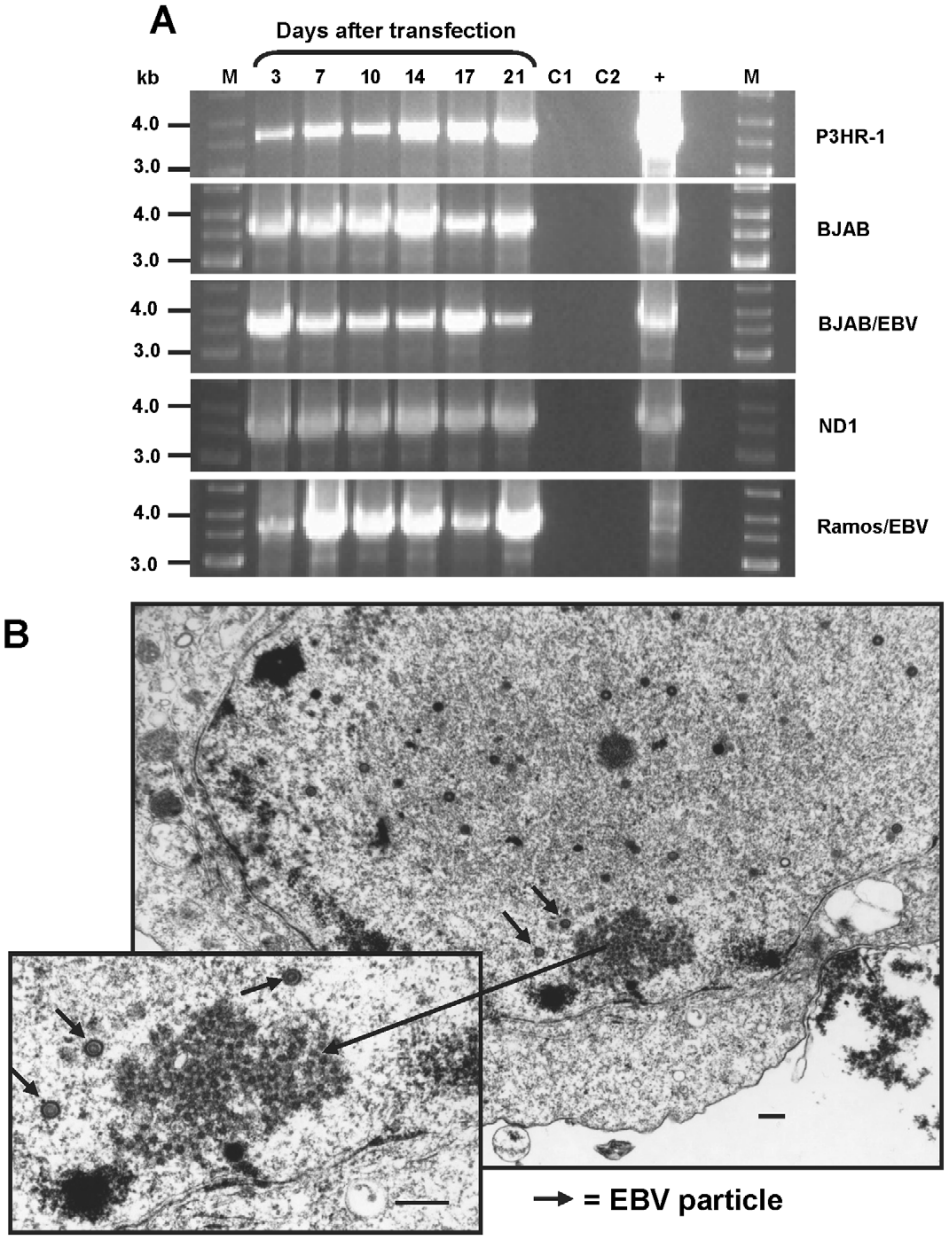
In vitro replication of TTV-HD14b and TTV-HD14c as measured by long-PCR amplification. (A) PCR amplification of full-length TTV-HD14b in P3HR-1, BJAB, BJAB/EBV and full-length TTV-HD14c in ND1 and Ramos/EBV cell lines. Days after transfection are indicated. M - DNA size marker, C1 - untransfected cells, C2 - untransfected cells with nucleofector solution, **+** - positive control for long PCR amplification consisted either of re-ligated TTV-HD14b or TTV-HD14c mixed with Ramos cell line DNA. (B) Electron micrograph of B95-8 cells 3 days after transfection with linearized TTV-HD14b DNA. TTV-like particles can be seen within the nucleus of a cell. EBV particles are indicated by short arrows. Bar = 250 nm.

We subsequently demonstrated the helper-dependent long-term replication and propagation of 12 TTV isolates in the 293TT cell line harbouring SV40 large T-antigen [67]. In the present study we analysed whether EBV is also able to exert a helper function for the replication of TTV. We included Burkitt's lymphoma cell lines both positive (P3HR-1, BJAB/EBV, Ramos/EBV) and negative for EBV (BJAB, Ramos), an EBV-immortalized B cell line (ND1) from an MS patient and an EBV-producing B cell line (B95-8). Replication of TTV-HD DNA was confirmed in all tested cell lines by long distance PCR on total cellular DNA. Long distance PCR was performed using back-to-back primers specific for amplifying the full-length genomes of TTV-HD14b and TTV-HD14c. Examples are presented in Figure 1A. Results were comparable between TTV-HD14b and TTV-HD14c and replication was measured up to day 21 after transfection in all cell lines. Replication was also confirmed by electron microscopy (Figure 1B).

### Replication of TTV-HD14b and TTV-HD14c is increased in the presence of EBV

Having demonstrated virus replication after transfection of TTV-HD14b- and TTV-HD14c-full-length genomes into EBV-positive and -negative cell lines, we quantified the level of TTV replication by real-time PCR. Viral DNA was normalized against the housekeeping gene HMBS and relative quantification was calculated using the EBV-negative cell line BJAB as calibrator. All measurements were performed for each time point when cells were harvested. We used two independent primer sets per isolate for the detection of both TTV-HD14b and TTV-HD14c. Two isolates of TTV-HD14 were included in this study in order to avoid possible variations which may result from differences in the TTV genome organization. Primers and probes are listed in Table 1.

**Table 1:** Primers and probes used.

Primers^a^	Sequence (5′-3′)^b^	Nucleotide position	Target
**Long-PCR**			
jt34f-7-F	5′-CAATTCGGGCTCGGGACTG-3′	216–234	TTV-HD14b
jt34f-8-R	5′-CCCCTTGACTGCGGTGTGTA-3′	215–196	TTV-HD14b
t3pb-1-F	5′-CAATTCGGGCACGGGACT-3′	216–233	TTV-HD14c
t3pb-2-R	5′-CCCCTTGACTTCGGTGTGAAACT-3′	215–192	TTV-HD14c
**In vitro transcription**			
gpU2-BamHI	5′-GCAGGATCCAGAATCTGGGCTGG GACGTT-3′	89585–89604	EBV gp350/220
gpL2-EcoRI	5′-GCAGAATTCACATGGAGCCCGGA CAAGT -3′	89784–89766	EBV gp350/220
**Real-time PCR**			
qP31-F	5′-ACAGACCAATCAGGACCTTCTAC-3′	31–53	TTV-HD14b/c
qP133-R	5′-CGGACGGGCGAAGAAAAAC-3′	133–115	TTV-HD14b/c
qP113-Pr	5′-FAM-CTACCATTCGTCCACCGCTGTT GCTT-3′- TAMRA	113–89	TTV-HD14b/c
qP326-F	5′-GTGCCAGGTAGAGGGAATCAATG-3′	326–348	TTV-HD14b/c
qP430-R	5′-GCGAGGAGCAATGCCGTTAAG-3′	430–410	TTV-HD14b/c
qP396-Pr	5 -FAM-TCACCACACCCGCAGAAAGCA GCAT-3′- TAMRA	396–372	TTV-HD14b/c
EBV-pol-F	5′-CTTTGGCGCGGATCCTC-3′	1978–1994	EBV BALF5
EBV-pol-R	5′-AGTCCTTCTTGGCTAGTCTGTTGAC-3′	2044–2068	EBV BALF5
EBV-pol-Pr	5′-FAM-CATCAAGAAGCTGCTGGCGGC C-3′- TAMRA	1998–2019	EBV BALF5
gpU2	5′-AGAATCTGGGCTGGGACGTT-3′	89585–89604	EBV gp350/220
gpL2	5′-ACATGGAGCCCGGACAAGT -3′	89784–89766	EBV gp350/220
EBVGPq	5′-FAM-AGCCCACCACAGATTACGG CGGT-3′- TAMRA	89761–89739	EBV gp350/220
HMBS-F	5′- CAGGACTAATYSAARTCTCTAC -3′	3731–3752	HMBS
HMBS-R	5′- CCAGAAAACTCACTGATTTCAA- 3′	3844–3823	HMBS
HMBS-Pr	5 -FAM-CTTGCTCGCATACAGACGGA CAGT-3′- TAMRA	3759–3782	HMBS

a - F, forward; R, reverse; Pr, probe.

b - Underlined letters indicate restriction sites.

**Figure 2:**
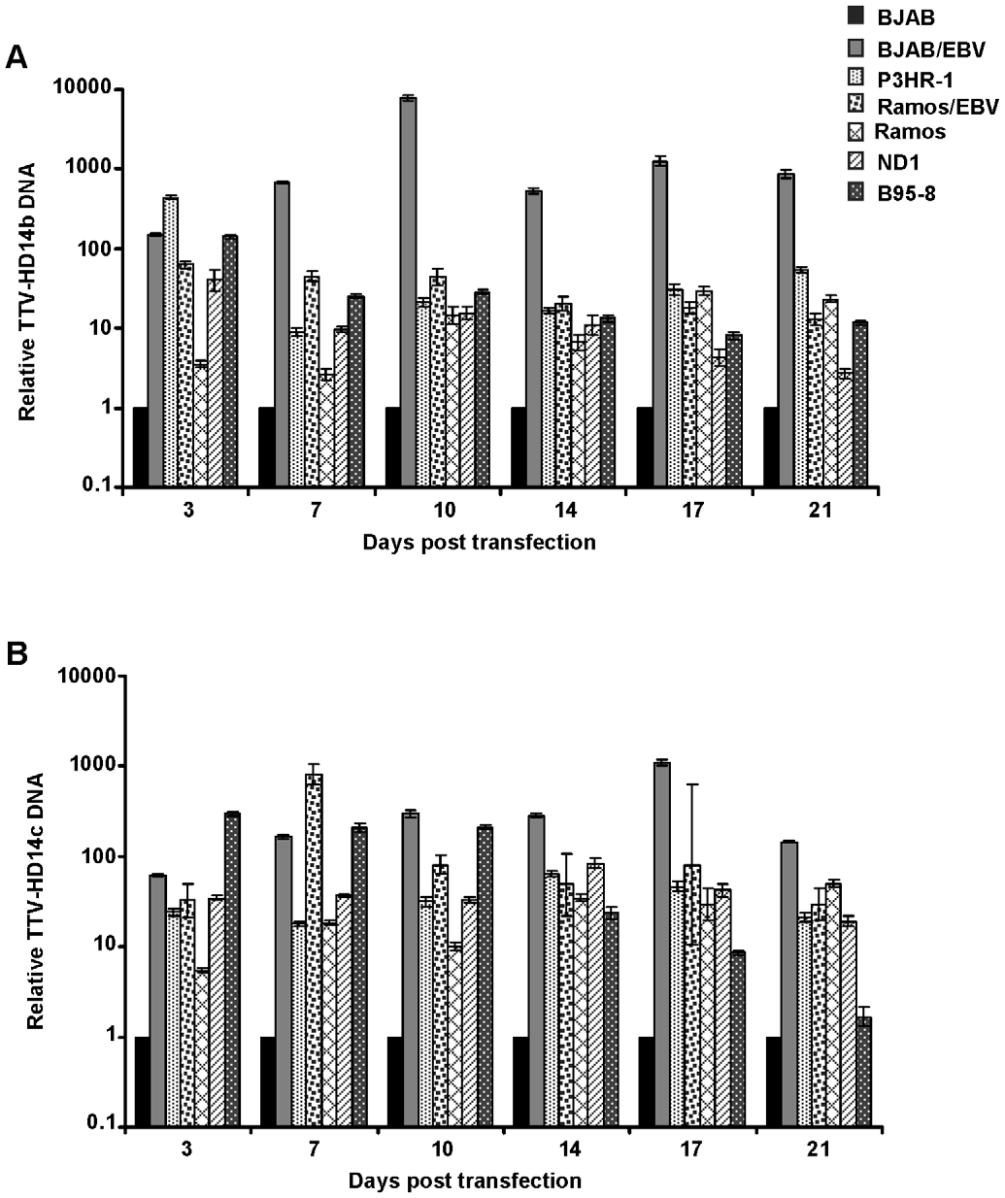
In vitro replication of TTV-HD14b and TTV-HD14c as measured by real-time quantitative PCR. Replication of (A) TTV-HD14b and (B) TTV-HD14c. qPCR values are expressed in ΔΔCt relative to BJAB (used as calibrator). Shown are mean ±95% confidence interval values of triplicate tests. A significant higher replication level of both TTV isolates was detected in all cell lines when compared to BJAB (p<%0.05).

The quantitative analyses performed with one set of primers and probe for TTV-HD14b and TTV-HD14c are shown in Figure 2 (A and B). Overall, TTV-HD replication was increased up to day 21 in all cell lines tested when compared to viral replication in BJAB. Comparable results were obtained with the second primer pair and probe selected for this study (data not shown) and a similar pattern of amplification was obtained with different biological replicates. Untransfected control and non-template control samples remained negative in all experiments. The presence of EBV in cell lines BJAB/EBV, P3HR-1 and Ramos/EBV resulted in elevated levels of TTV-HD14b and TTV-HD14c DNA replication. Replication of both TTV-HD14 isolates was significantly higher (p<0.05) in all cell lines when compared to BJAB at each time point of evaluation (Figure 2). From day 7 onwards, a higher level of TTV-HD14b replication (Figure 2A) was noted in the BJAB cell line converted to EBV-positivity, than in the other cell lines. Viral levels seemed to increase early after transfection, after which it levelled off. Replication of TTV-HD14b versus TTV-HD14c varied significantly (p<0.05) in each cell type, except for day 17 after transfection where replication differences between isolates in Ramos/EBV and B95-8 were not statistically significant, as well as day 3 where replication of the two TTV isolates did not differ significantly. TTV-HD14c replication in ND1 (EBV-immortalized B cells from an MS patient) (Figure 2B) seemed to be stable over time in comparison to a decrease for TTV-HD14b replication in these cells. An initial increase in B95-8 decreases more rapidly for TTV-HD14c than for TTV-HD14b. TTV replication behaviour in the EBV-positive Ramos/EBV cell line was surprising. The increased TTV-HD14b replication in Ramos/EBV versus Ramos cells was statistically significant (p<0.0001) up to day 14, where after TTV-HD14b replication in Ramos cells exceeded that in Ramos/EBV cells (days 17 and 21). TTV-HD14c replication in Ramos/EBV cells increased from day 3 to day 17, with levels reaching statistical significance for day 3 to 7 (p<0.0001), but not for days 14 and 17. Replication on day 21 was similar in both isolates with higher replication in Ramos than in Ramos/EBV cells. A previous report did not detect any difference in the level of permissiveness for an HSV-1 infection between Ramos and EBV-converted Ramos/EBV cells [74].

### The enhanced replication of TTV-HD14b and TTV-HD14c in the presence of EBV is independent of the EBV copy number

**Figure 3:**
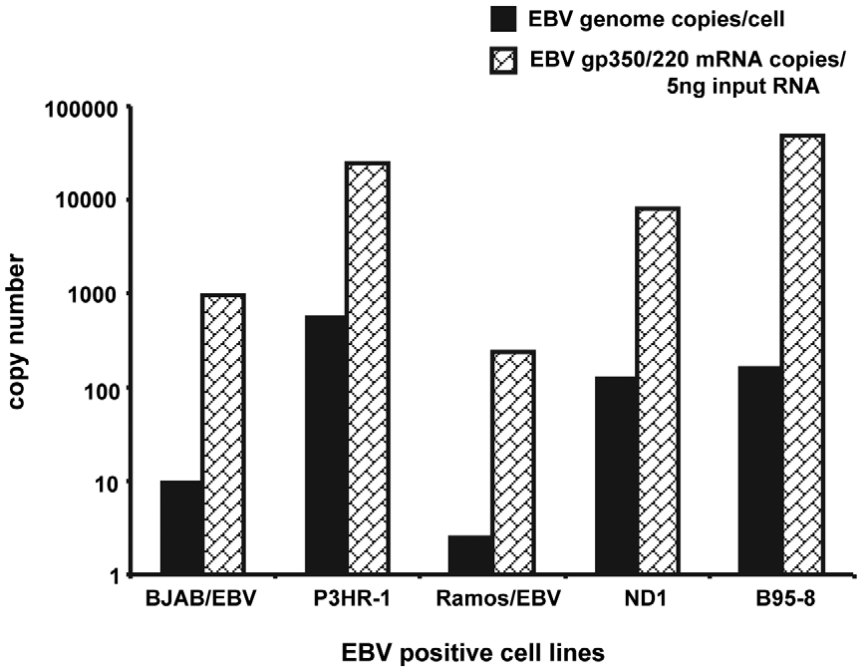
Quantification of EBV-genomes and -replication. Real-time qPCR in the EBV positive cell lines transfected with TTV-HD14c (day 14 post-transfection) was applied to determine the EBV genome copies per cell, as well as EBV replication as measured by gp350/220 mRNA.

Having measured varying levels of TTV replication in the respective EBV positive cell lines, we quantified the EBV DNA to determine whether EBV copy number exerted an influence. Real-time quantitative PCR was performed at all time points (day 3–21 post transfection) and in all cultures transfected with both TTV genomes using DNA from an EBV-harbouring (2 copies/cell) cell line Namalwa as standard curve [75]. A representative example of EBV copy number/cell at day 14 post-transfection of TTV-HD14c is presented in Figure 3. The EBV copy number in the P3HR-1 cell line was 583 copies/cell, whereas B95-8 cells harboured 161 EBV copies/cell. Interestingly, BJAB/EBV cells habor only 10 EBV copies/cell despite the high levels of TTV amplification. The lowest EBV copy number was found in Ramos/EBV containing from 2 EBV copies/cell. From this analysis we can conclude that although the presence of EBV seems to enhance replication of TTV-HD DNA, the EBV viral copy number does not reflect the differences in replication level between cell lines.

### EBV production measured by gp350/220 expression is consistent with the EBV copy number and does not correlate with TTV replication

Quantification of EBV DNA genome does not provide information regarding the intracellular activity of the EBV DNA. P3HR-1 and B95-8 are well-characterized cell lines in which EBV production has been reported [76], [77], whereas EBV viral capsid antigen (VCA) and synthesis was demonstrated in the BJAB/EBV cell line [78]. The actual activity of the EBV DNA is less clear in the Ramos/EBV cell line and unknown for the ND1 cell line. We therefore quantified the EBV envelope glycoprotein gp350/220 mRNA as marker for EBV lytic activity in all EBV positive cell lines used in this study [79]. The results obtained showed a similar pattern as the EBV genome quantification. For comparison of the EBV DNA copy number, the RNA quantification presented in Figure 3 represents day 14 after transfection of TTV-HD14c. B95-8 and P3HR-1 showed the highest EBV-gp350/220 copy numbers of 48268 and 25018 copies/5 ng of total cellular RNA, respectively (Figure 3). ND1 cells harboured 8141 EBV-gp350/220 copies/5 ng RNA, followed by BJAB/EBV and Ramos/EBV with 965 and 240 copies/5 ng total RNA, respectively. Consistent results were obtained with both TTV isolates and with different biological replicates. Thus, EBV was replicating (as measured by gp350/220 gene transcription) in all EBV-positive cell lines used in this study, but this productivity did not necessarily relate to the EBV copy number/cell. The level of this EBV activity however does not seem to influence the level of TTV-HD replication.

## Discussion

We have transfected the full-length genomes of two TT viruses isolated from brain tissue from an MS patient in order to ascertain first, whether viral replication takes place in different B lymphocytic and Burkitt's lymphoma cell lines and, secondly, to determine whether EBV acts as a helper virus for the replication of TTV. The results presented here demonstrate replication of both TTV-HD14b and TTV-HD14c up to day 21 post-transfection. Overall, we observed that the replication pattern of both TT viruses is cell-type dependent with higher levels of replication measured in the EBV positive cell lines, but showing variability in the ΔΔCt at certain time points (Figure 2). A number of studies have demonstrated the ability of herpesviruses to induce amplification of other persisting viral DNA [80]. Several lines of evidence may indicate the mechanism through which EBV exerts its helper-function for TTV replication. Our previous study [67] demonstrated a requirement for a helper-function in the replication and propagation of a series of TTV isolates in the 293TT cell line which had been constructed to express high levels of SV40 large T-antigen [81]. The putative origin of replication in TT viruses harbours a number of pentanucleotide motifs very similar to binding sites for SV40 large T-antigen. EBNA-1 is required for the episomal maintenance of EBV DNA and binds to the origin of replication in a sequence-specific manner [82]. Cellular transcription and gene expression is also regulated by EBNA-1 through binding to cellular promoters. Analyses of a large number of these promoters indicated differences in EBNA-1 binding motifs between those found in the EBV genome and cellular genome [83], indicating a notion of additional, yet unidentified, binding sites for EBNA-1. Cellular promoters devoid of EBNA-1 binding sites may even be upregulated by the mere presence of EBNA-1 in the cell [84]. Enhancement of HCV replication by EBNA-1 via a transactivating function has been demonstrated [73]. Additional investigation is needed to clarify the mechanism through which EBNA-1 may act to stimulate the TTV genome. We selected several EBV-positive Burkitt's lymphoma- and B cell lines in order to evaluate the TTV-HD14b and TTV-HD14c replication. All these EBV-positive cell lines contained the EBNA-1 as evidenced by the quantitative measurement of the envelope glycoprotein gp350/220 mRNA as marker for EBV lytic activity (Figure 3). The TTV origin of replication is present in both TTV-HD14 isolates used in the present study and was not influenced by other differences between these 2 viral genomes [this study, 67].

A role for virus infections in the etiology of multiple sclerosis has repeatedly been investigated [4], [36]. A possible role for TTV infection in autoimmune disease including multiple sclerosis, has been reported previously [43], [52], [61]–[63]. The role of EBV infections in MS has however been controversial ranging from failure to demonstrate any presence [38]–[40], [85] to demonstration of an increased risk for a previous EBV infection [9], [86] and elevated EBV antibodies, more specifically EBNA-1 antibodies in MS patients [4], [87]–[91].

Presently, studies on an association between both TTV and EBV infection in lymphocytes of B-cell lymphoma and Hodgkin's lymphoma have been inconclusive [92], [93]. An increased permissiveness for infection with herpes simplex virus during the first 48 hours of infection has been demonstrated in EBV-converted, previously negative Burkitt's lymphoma derived cell lines [74]. Therefore the presence of both TTV and EBV in the same B cell is feasible. Naive and memory B cells are equally susceptible to EBV infection [94]. EBV infection may lead to EBV persistence in B cells providing ground for differentiation into EBV-latently infected circulating memory B cells [95]. Ectopic follicle-like structures harbouring B cells, similar to those found in other organ-specific immune disease, have also been described in the cerebral meninges of some MS patients [96]. The persistence of EBV infection, as measured by elevated EBNA-1 antibodies, could provide a fertile field for the induction of molecular mimicry. It has been proposed that autoreactive T cells induced by molecular mimicry may need such a fertile field of persistent viral infection to become autoaggressive [97]. *In vivo* clonally expanded CD4^+^T cells isolated from cerebrospinal fluid of an MS patient, responded to a poly-arginine motif present in TT viruses [43]. The TTV-HD14 isolates used in the present study were isolated from MS brain tissue. Putative proteins from genes and their transcripts of TTV-HD14 and its μTTV share similar but sufficiently modified signature motifs to cellular genes including myelin basic protein (MBP) (unpublished results). A number of studies have indicated an elevated myelin specific T cell population in MS patients [36], [47]. We propose the following model for a role of both EBV and TTV infections as etiological agents in MS: Simultaneous infection by either TTV or μTTV in EBV-infected memory B cells leads to persistence of the TTV supported by EBNA-1 expression. Spontaneous activation of the EBV lytic cycle in such cells could result in a burst of TTV or μTTV expression and production and infection of neighbouring cells. TTV autoimmune-reactive epitopes in turn induce autoreactive T cells [97] reacting against specific proteins of the TTV-infected cells. This model could explain the local autoimmune response, the focal nature of MS lesions and the re-emergence of new foci. We have demonstrated EBV-helper dependent activation of TTV-HD14 isolated from MS brain tissue. Ongoing studies are investigating the cross-reactivity of TTV-HD14 expressed proteins to myelin immune response.

## Materials and Methods

### Cell culture

Seven hematopoietic cell lines, either EBV-positive or EBV-negative, were selected for this study. Burkitt's lymphoma cell lines included BJAB and Ramos, both EBV-negative [98], [99] and P3HR-1 harboring an EBV genome in which the EBV nuclear antigen-2 (EBNA-2) has been deleted [76]. For comparison we included the BJAB and Ramos cell lines which had previously been converted to EBV-positive by infection with the EBV genome originating from the P3HR-1 cell line, BJAB/EBV and Ramos/EBV [98], [100]. In addition, two B cell lines were included. A human EBV-immortalized B cell line ND1, established from an MS patient (kind gift from M. Sospedra) and the EBV-producing B95-8 cotton-top marmoset cell line [77]. EBV DNA was quantified using serial dilutions of Namalwa DNA (human Burkitt's lymphoma cell line containing 2 EBV genome copies per cell, kindly provided by Regina Feederle) as reference for a standard curve [75]. All cell lines (mycoplasma free as confirmed by PCR) were maintained in RPMI 1640 medium supplemented with 10% fetal calf serum and 1% penicillin-streptomycin in 5% CO_2_ at 37°C.

### TTV genomes

Thirty full-length TTV genomes were isolated in our laboratory from 4 brain samples [67], the majority differing in their genome organization from that described for these viruses [49]. The reason or consequence of these rearrangements in TT viruses is to date not known [52], [67]. We therefore selected 2 full-length genomes originating from one brain sample for the transfection assays. The ORF1 of TTV-HD14b (accession number FR751464) is fused with the last 37 nt of ORF4 and part of the control region, whereas TTV-HD14c (acc. no. FR751465) ORF1 is fused to ORF5. Viral genomes were released from the pCR2.1 vector construct (Invitrogen) and purified after separation by gel electrophoresis (PeqLab Biotechnologies) and prior to transfection of cells.

### Transfection of cells and DNA/RNA processing

Transfection of 2 µg TTV-DNA into 5×10^6^ cells was performed by using electroporation (Nucleofector II transfection device, Amaxa Biosystems) and the Nucleotransfection kit V (cat# vco-101 Amaxa Biosystems) and following protocols recommended by the manufacturer for each respective cell type. Transfection efficiency was controlled for by parallel transfections with the plasmid pmaxGFP (Amaxa Biosystems). Negative controls of each cell line with and without nucleofector solution were included. Cells were transfected for harvesting on days 3, 7 and 10, whereas transfected cells harvested at days 14, 17 and 21 were passaged from transfected cultures for day 7, 10 and 14, respectively. Cells were passaged at 7 day intervals and fresh medium added when cells became too dense during this interval. Transfection assays were performed in parallel by 2 of us (S.B. and C.W.). Cultures were harvested on days 3, 7, 10, 14, 17 and 21 after transfection at an average density of 1–1,5×10^6^/ml and total DNA and RNA isolated. Total DNA was extracted using phenol-chloroform-isoamylalcohol [65] and subjected to DpnI (Fermentas) digestion to remove input DNA used for transfection. Total RNA was isolated from the transfected cells using the RNeasy mini kit (Qiagen, Hilden, Germany). All RNA samples were treated with DNaseI (Promega) to remove any residual DNA. The quality of the RNA samples was checked by running the samples in a 1% agarose gel.

### PCR amplification of full-length TTV genomes

In order to investigate whether replication of the transfected TTV-HD14b and TTV-HD14c occurred in the tested cell lines, the genomic DNA isolated from all transfected cell lines was further subjected to long-PCR amplification using TaKaRa LA Taq enzyme (TAKARA BIO INC) and back-to-back primers. The specific primers used for each individual TTV type are documented in Table 1. The reaction was performed as follows: initial denaturation for 1 min at 94°C, followed by 30 cycles of 30 s at 94°C, 1 min at 68°C (TTV-HD14b) or 1 min at 65°C (TTV-HD14c) and 4 min at 72°C, followed by final elongation at 72°C for 10 min.

### TTV and EBV detection by real-time quantitative PCR

Two sets of primers and the respective hydrolysis probes were designed on the TTV-HD14b and TTV-HD14c genomic region spanning nucleotide 1 to 430 (identical between isolates). Primers and probes for both TTV-HD14b and TTV-HD14c were obtained from Biomers.net. The two sets (qP31F-qP133R-qP113Pr and qP326F-qP430R-qP396Pr) were separated by 192 nucleotides. Genomic DNA samples (100 ng in 5 µl) were amplified in triplicate, each in a total volume of 20 µl containing 12.5 µl Taqman Universal master mix (Applied Biosystems), 0.5 µl forward primer (10 µM), 0.75 µl reverse primer (20 µM), 0.63 µl 5′FAM-3′TAMRA-labelled probe (10 µM) and 0.62 µl water. Reaction mixtures were amplified for 40 cycles (15 s at 95°C and 1 min at 60°C) after an initial activation of the DNA polymerase for 10 min at 95°C. Fluorescent signals were detected in an ABI 7300 sequence detection system (Applied Biosystems). Results for each time point (day 3, 7, 10, 14, 17 and 21) were standardized against the amplification of TTV-HD14b and TTV-HD14c in the EBV-negative BJAB cell line and expressed as fold change values. DNA samples obtained from untransfected cells, as well as non-template controls (NTC) were included in each reaction plate.

Data normalization was performed using the HMBS (hydroxymethylbilane synthase) housekeeping gene reported to share the same specificity in both human and cotton-top tamarin B95-8 cell lines [101]. Beacon designer 7.9 software was used for the design of the Taqman assay for HMBS, selecting a homologous region between the human and cotton-top tamarin genome. Validation of the primers and probes and assay optimization were performed as described previously [67]. Amplification reactions of HMBS were performed in 25 µl volumes, containing 12.5 µl Taqman Universal master mix (Applied Biosystems), forward and reverse primers 0.75 µl each (10 µM), 0.75 µl 5′FAM-3′TAMRA-labelled probe (10 µM) and 5.25 µl water. The AmpliTaq Gold DNA Polymerase was activated at 95°C for 10 min prior to amplification through 40 cycles (15 s at 95°C and 1 min at 60°C) during which fluorescent signals were measured.

EBV DNA was quantified by targeting the BALF1 gene [75] in the EBV positive cell lines. Genomic DNA samples (2 ng) in triplicates were used for the reaction and NTC included in each reaction plate. Primers and probes used to detect EBV DNA are listed in Table 1. Quantification of EBV DNA in each cell line was calculated against a standard curve generated by serial dilutions of Namalwa DNA (2 copies of EBV DNA per cell) as previously described [75].

EBV activity/replication was measured by quantifying mRNA production of a 200 bp fragment of the EBV envelope glycoprotein gene gp350/220 [79]. Primers and a hydrolysis probe used are listed in Table 1. The RNA samples (5 ng) from the transfected cells were amplified using a one step RT-qPCR protocol (Applied Biosystems). Briefly, the RT-qPCR mixture contained: 12.0035 µµl of Taqman Fast Virus 1-Step Master Mix, 1.5 µl each of forward and reverse primers (10 µM), 1.5 µl of 5′FAM-3′TAMRA-labelled probe (10 µM), 28 µl water and 5 µl RNA. An initial reverse transcription step (5 min at 50°C), was followed by RT inactivation/initial denaturation (20 s at 95°C). Reaction mixtures were subsequently amplified for 40 cycles (15 s at 95°C and 1 min at 60°C). Negative controls included NTC and samples without reverse transcriptase. Fluorescent signals were detected using an ABI 7300 sequence detection system (Applied Biosystems). EBV gp350/220 gene expression was quantified using an external RNA calibration standard which was generated as follows: The EBV late envelope glycoprotein gp350/220 gene was transcribed from B95-8 cellular RNA with SuperScript II reverse transcriptase (Invitrogen) using specific primers containing restriction sites for BamHI and EcoRI (Table 1). The resulting 200 bp cDNA amplicon was cloned into the pSPT18 vector (SP6/T7 Trancription Kit, Roche) as described previously [79]. Sequence analysis verified the sequence. The cDNA-containing plasmid was linearized with EcoRI prior to subsequent *in vitro* transcription using SP6 RNA polymerase (SP6/T7 Trancription Kit, Roche). The size and quality of this transcript were determined by denaturing RNA electrophoresis. Serial dilutions (5-fold) of this 200 bp gp350/220 transcript in 5 ng BJAB RNA (EBV negative) in DNase/RNase free water were used to calibrate the EBV gp350/220 gene expression. Standards were stored in aliquots at −70°C for no longer than 1 week and each aliquot was used once.

### Statistical analysis

TTV-HD14b and TTV-HD14c amplification in the BJAB cell line (EBV-negative) was used as a calibrator to compare TTV amplification between cell lines. For these analyses the mean ΔΔCt was derived in each experiment and the 95% confidence interval for mean calculated. These values were converted to fold changes. Comparisons between TTV-HD14b and TTV-HD14c amplification levels (fold changes) in each of Ramos (EBV-negative) and Ramos/EBV cell lines were performed by t-test.
